# Probable Primary Cutaneous Cryptococcosis in an Immunocompetent Man after Contact with Wild Bird Droppings

**DOI:** 10.4269/ajtmh.24-0646

**Published:** 2025-05-20

**Authors:** Felipe Tavares Rodrigues, Priscilla Filippo Alvim de Minas Santos, Regina Casz Schechtman

**Affiliations:** Dermatology, Universidade do Estado do Rio de Janeiro, Rio de Janeiro, Brazil

**Figure 1.**
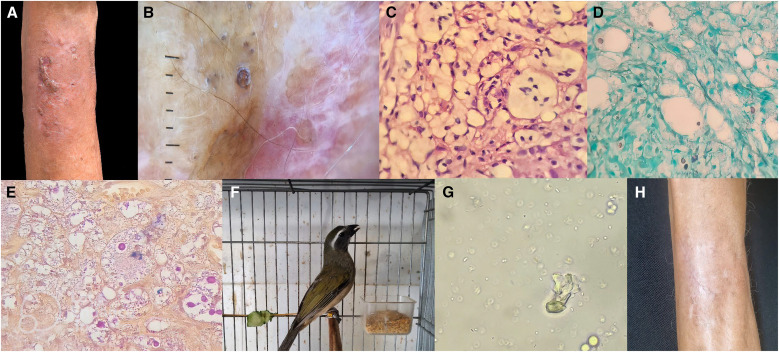
(**A**) Skin lesion aspect. (**B**) Dermoscopy aspect with erythema, white fibrosis strains, and disease activity areas that resemble blackheads. (**C**) Hematoxylin and eosin stain biopsy showing numerous histiocytes intracytoplasmatic yeasts (×10 magnification). (**D**) Grocott–Gomori stain showing the silver-positive birefringent yeasts. (**E**) Histological sections stained with mucicarmine showing the birefringent yeasts. (**F**) Trinca-ferro bird specimen. (**G**) Direct mycological examination showing *Trichosporon* sp. (**H**) Improvement of the lesion after treatment.

A 65-year-old man, a smoker with obstructive pulmonary disease, developed an erythematous infiltrating skin lesion with a scarring, hyperkeratotic crust that had been evolving for 5 months (Figure 1). A skin biopsy with histopathology indicated large numbers of yeast-like organisms surrounded by mucinous capsular material forming mucoid masses in the dermis. The mucinous capsular materials were stained red and black with mucicarmine and Grocot–Gomori silver stain, respectively (Figure 1). The patient raised a caged bird, a green-winged saltator (Figure 1). Reportedly, before the lesion appeared, he had suffered insect bites at the infection site. After scratching the site, a skin injury occurred and possibly came in contact with bird excreta when cleaning the cage. Direct light microscopic examination of fecal samples in the cage showed yeast-like structures that could represent specimens of *Cryptococcus* sp. Only *Trichosporon* spp. grew (Figure 1).

We ruled out the risk of hematogenous dissemination after a negative blood culture and an unremarkable chest X-ray. Fungal culture of the biopsy sample was negative. After surgically excising most of the lesion and administering fluconazole (150 mg for 15 weeks by mouth), the lesion resolved by 6 months (Figure 1).

We highlight that the cutaneous cryptococcosis does not necessarily affect patients with suppressed immune systems and that it can behave like a subcutaneous mycosis caused by primary inoculation, such as sporotrichosis and chromoblastomycosis. We emphasize the importance of obtaining a comprehensive history that includes contact with birds as a possible epidemiological factor.^1^^–^^3^
